# Supplementary figures and images for: Biogenesis of P-TEFb in CD4+ T cells to reverse HIV latency is mediated by protein kinase C (PKC)-independent signaling pathways
Source: PLoS Pathog. 2021 Sep 16;17(9):e1009581. doi: 10.1371/journal.ppat.1009581 (PMC8478230; doi:10.1371/journal.ppat.1009581)

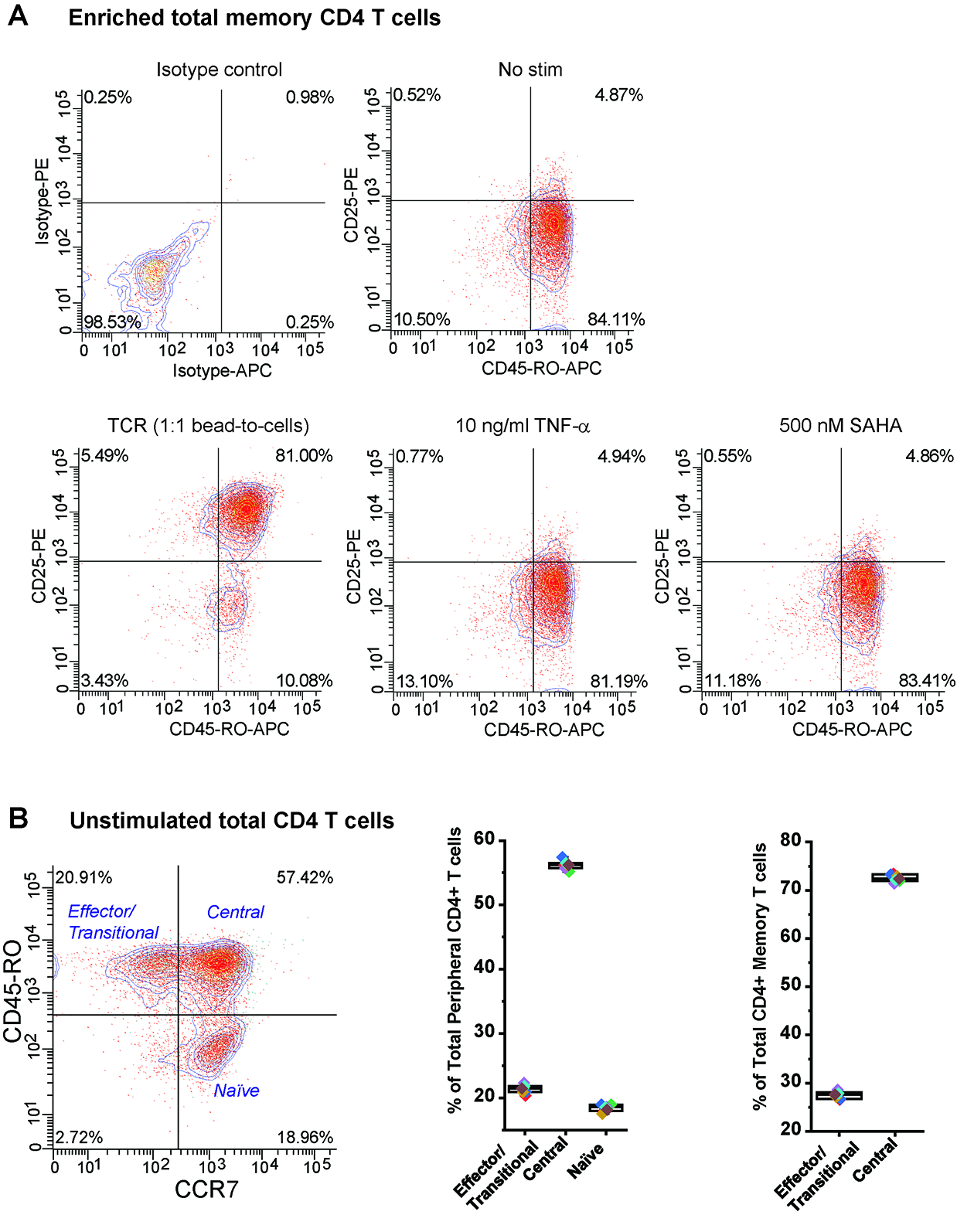

Supplement: S1 Fig — (A) Surface phenotype of total memory CD4+ T cells isolated using the EasySep Memory CD4+ T Cell Enrichment Kit (Cat. # 19165). Following memory CD4+ T-cell isolation cells were treated or not for 24 h with the stimuli shown then subjected to immunofluorescence flow cytometry after staining with fluorophore-conjugated isotype controls or antibodies towards CD25 and CD45-RO. (B) Central memory CD4+ T cells account for the majority of both total peripheral CD4+ and memory CD4+ T cells. Total peripheral CD4+ T cells were isolated from healthy donor PBMCs using EasySep Human CD4+ T Cell Kit (Cat. # 17952). Thereafter, cells were subjected to immunofluorescence flow cytometry after staining with fluorophore-conjugated antibodies towards CCR7 and CD45-RO. Based on this dual staining, CD4+ T-cell subsets were determined in multiple experiments as a fraction of total peripheral CD4+ or total memory CD4+ T cells. (TIF) [file ppat.1009581.s001.tif]

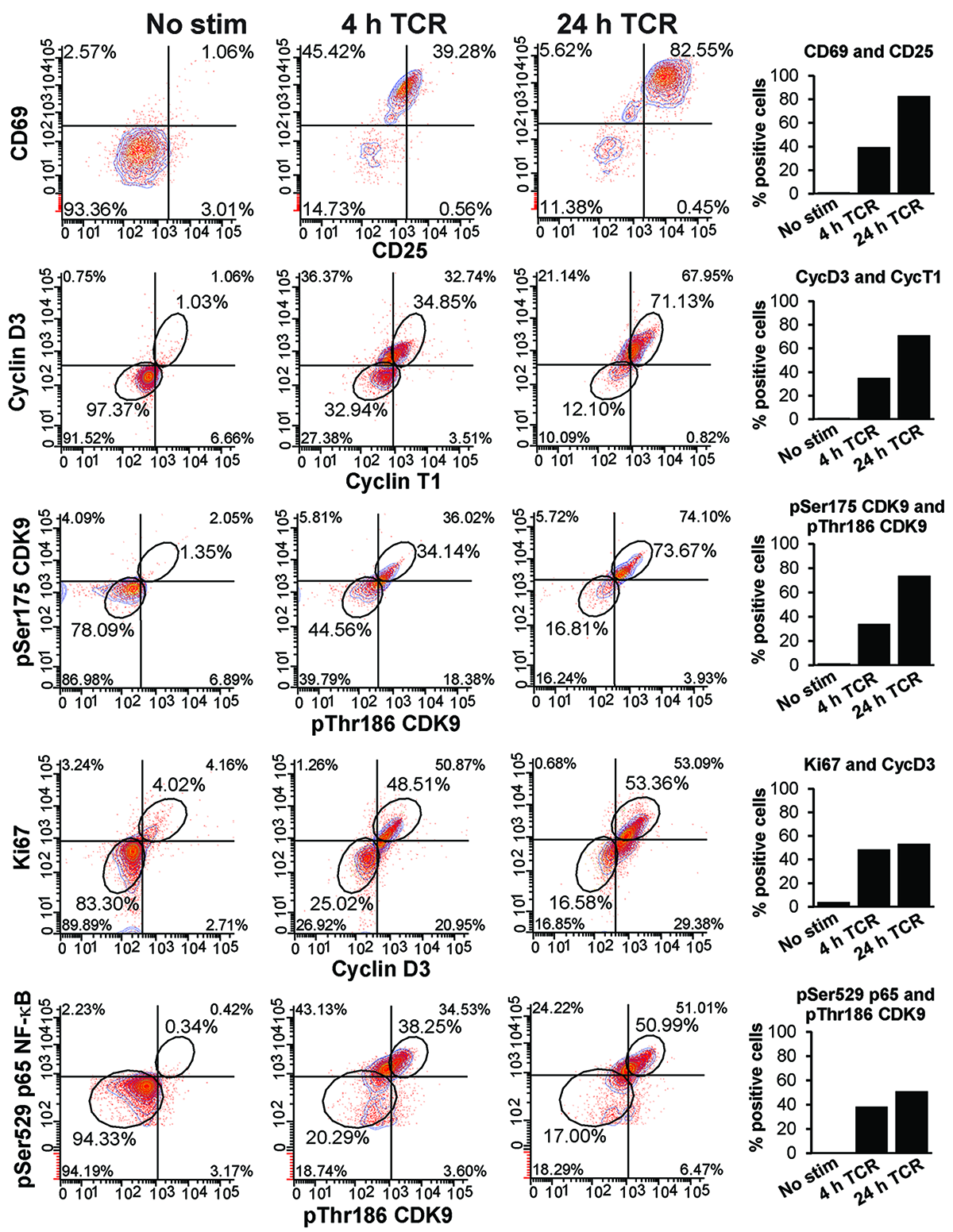

Supplement: S2 Fig — Untreated and 4 h or 24 h TCR-activated memory CD4+ T cells were subjected to immunofluorescence flow cytometry to monitor the expression of CycT1, pSer175 or pThr186 T-loop phosphorylated CDK9, the activated form of NF-κB (pSer529 p65 NF-κB), T-cell proliferative markers Ki67 and cyclin D3, and the T-cell activation surface markers CD25 and CD69. The flow cytometry data in each panel are summarized in the bar graphs shown to the right. (TIF) [file ppat.1009581.s002.tif]

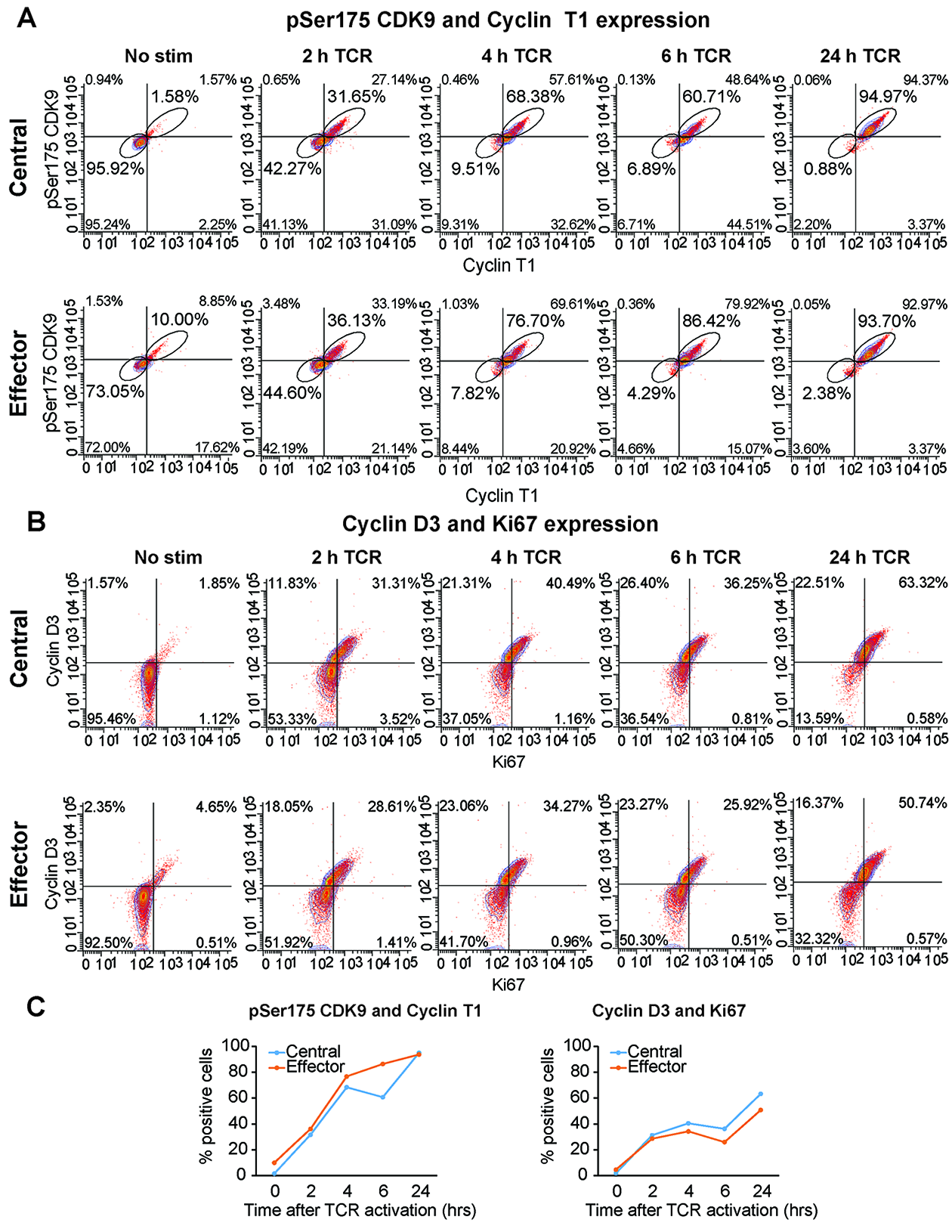

Supplement: S3 Fig — (A) and (B) Following purification of central and effector memory subsets from healthy donor PBMCs using the EasySep Human Central and Effector Memory CD4+ T Cell Isolation Kit (Cat. # 17865), they were stimulated through the TCR with anti-CD3/anti-CD28 Dynabeads for varying times as shown. Thereafter, cells were subjected to immunofluorescence flow cytometry to monitor the expression of the P-TEFb (pSer175 CDK9 and CycT1) or Ki67 and cyclin D3. (C) Graphical representation of the flow cytometry data shown in A and B. (TIF) [file ppat.1009581.s003.tif]

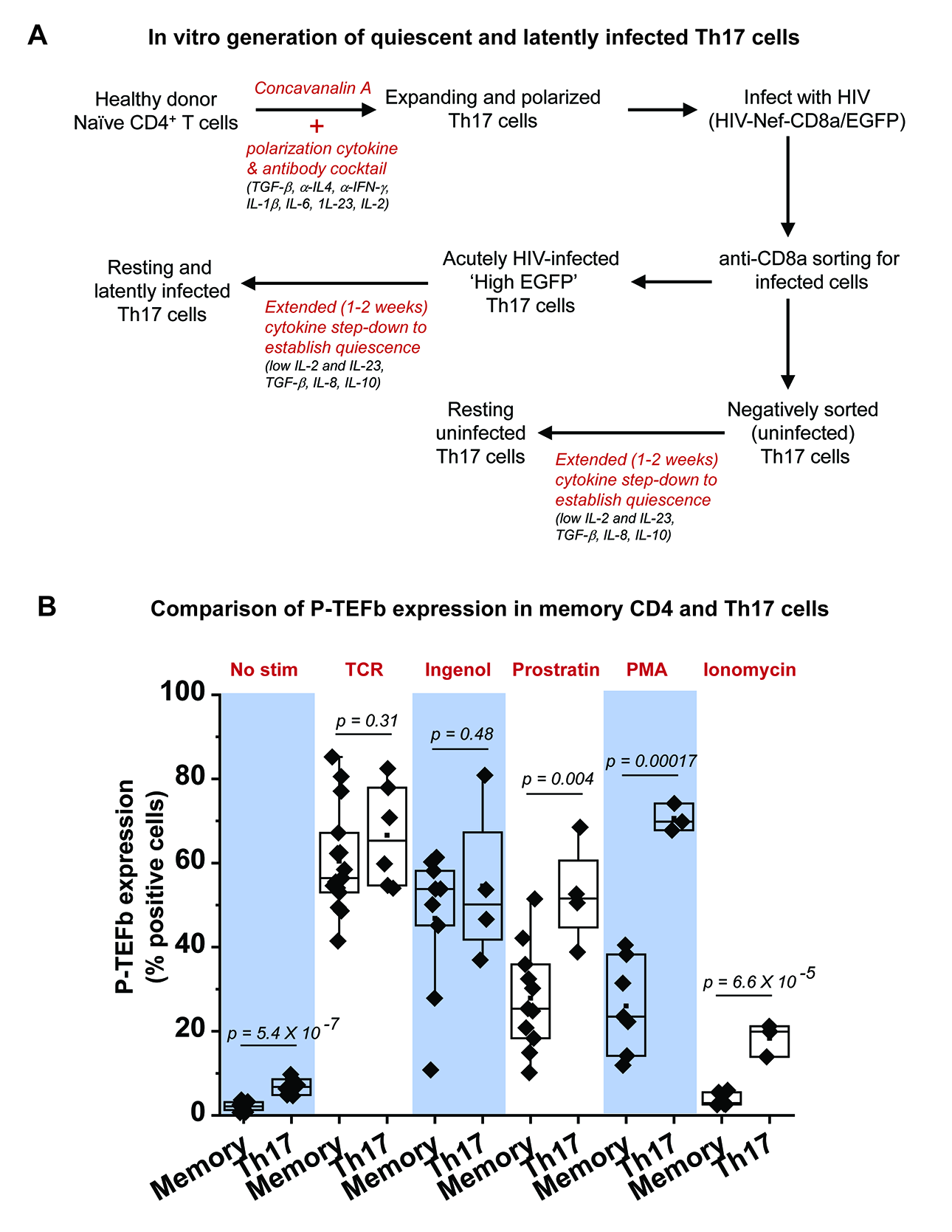

Supplement: S4 Fig — (A) Procedure for generating polarized quiescent primary Th17 cells from healthy donor-derived naïve CD4+ T cells, with or without a latent infection with HIV. (B) Direct comparison of the extent of P-TEFb expression in memory CD4 T cells and primary Th17 cells using the data shown in Fig 1D. P-TEFb expression is measured as the dual expression of CycT1 and pSer175 CDK9. Statistical significance (p values) was calculated using a two-tailed Student’s t test. (TIF) [file ppat.1009581.s004.tif]

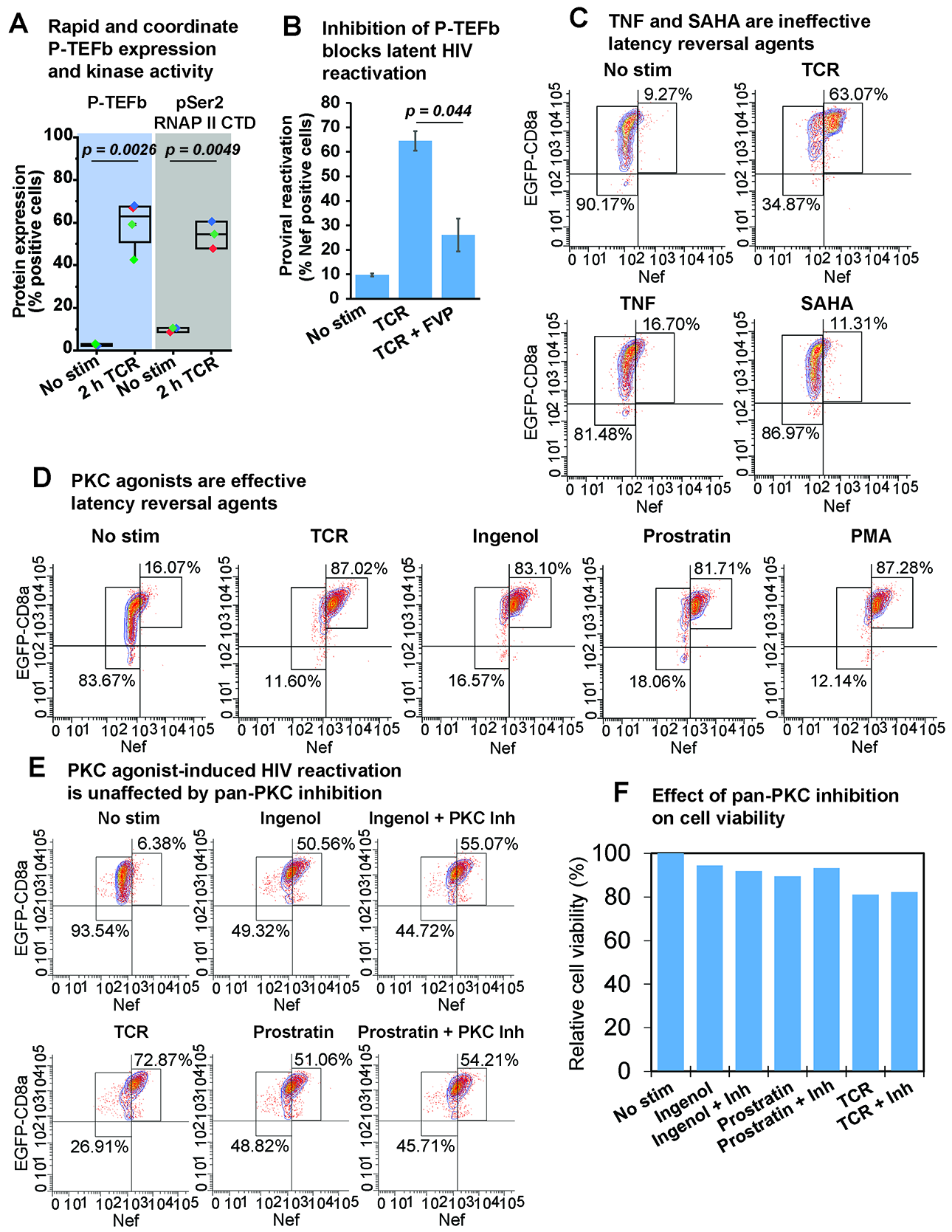

Supplement: S5 Fig — (A) Rapid expression of kinase active P-TEFb in memory CD4+ T cells in response to TCR co-stimulation. Memory T cells from three different healthy donors (color-coded) were activated or not for 2 h through the TCR with anti-CD3/anti-CD28 Dynabeads. Afterwards, the cells were subjected to flow cytometry analysis following intracellular immunofluorescence staining for P-TEFb (by co-staining for CycT1 and pSer175 CDK9) or the C-terminal domain Ser2 phosphorylated form of RNA polymerase II (pSer2 RNAP II CTD). (B) A selective inhibitor of CDK9 kinase, flavopiridol (FVP) effectively blocks TCR-mediated proviral reactivation in the QUECEL primary Th17 model of HIV latency. Latently infected Th17 cells were treated or not with 100 nM FVP for 30 min prior to TCR co-stimulation with anti-CD3/anti-CD28 Dynabeads for 24 h. Thereafter, cells were analyzed by flow cytometry following immunostaining using a fluorophore-conjugated antibody towards the HIV Nef accessory protein. Error bars denote S.E. of the mean from three separate experiments. Statistical significance (p values) in both A and B was calculated using a two-tailed Student’s t test. (C) TNF-α and SAHA are inadequately able to reactivate latent HIV in primary Th17 cells. Cells were challenged with anti-CD3/anti-CD28 Dynabeads (1:1 bead-to-cells), 10 ng/ml TNF-α or 500 nM SAHA for 24 h. Proviral HIV expression was examined by immunofluorescence flow cytometry following immunostaining using a fluorophore-conjugated antibody towards HIV Nef. (D) Ingenol, prostratin and PMA can sufficiently reactivate latent HIV in primary Th17 cells. Cells were challenged with anti-CD3/anti-CD28 Dynabeads (1:1 bead-to-cells), 50 nM ingenol, 1 μM prostratin or 50 ng/ml PMA. Proviral HIV expression was examined by immunofluorescence flow cytometry following immunostaining using a fluorophore-conjugated antibody towards HIV Nef. (E) Representative flow cytometry data showing that a combination of two PKC inhibitors (Ro-31-8220 and Gö 6983) [file ppat.1009581.s005.tif]

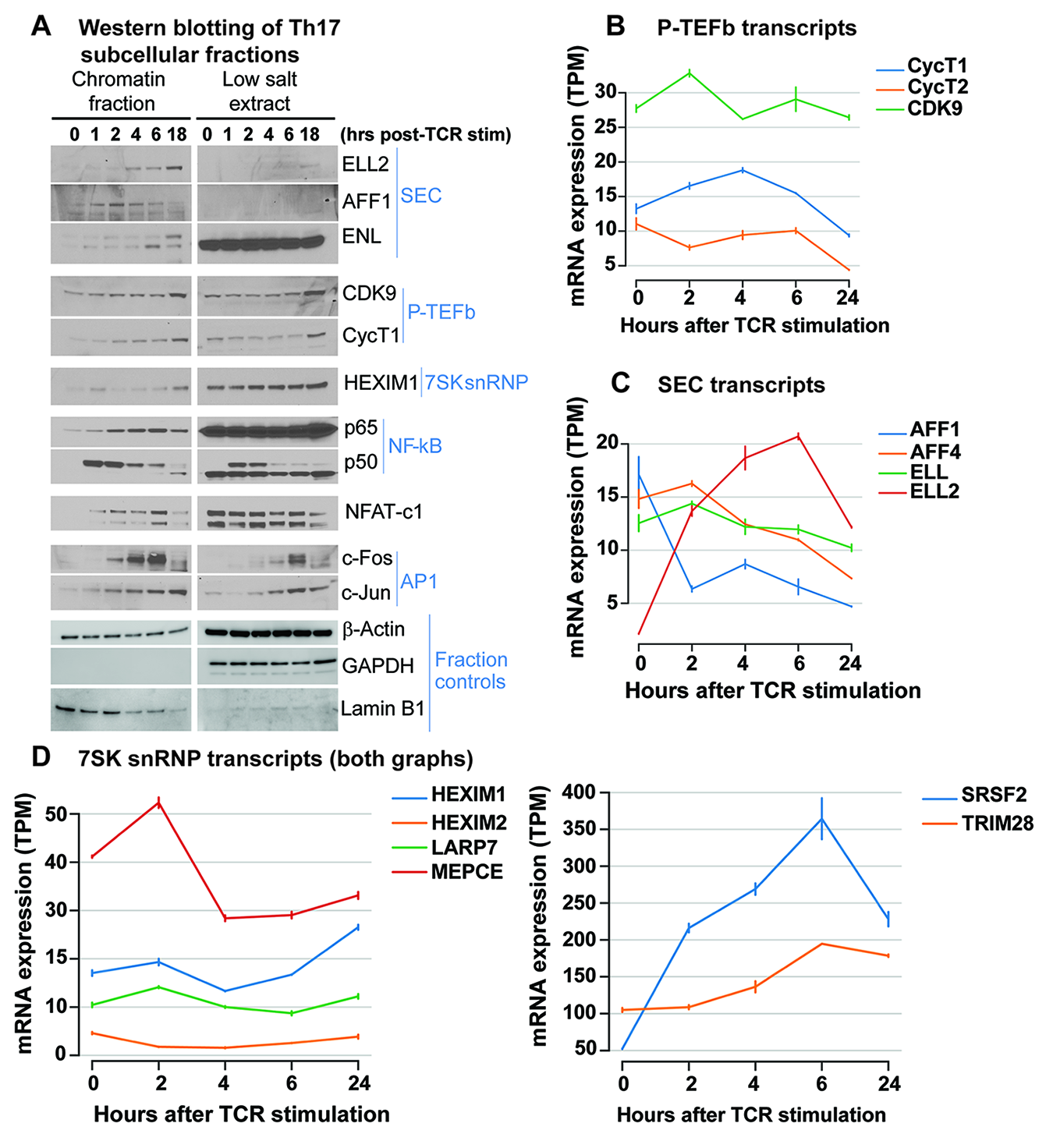

Supplement: S6 Fig — (A) Immunoblotting analysis of resting and TCR-activated primary Th17 cells to examine the expression of P-TEFb subunits (CDK9 and CycT1), its 7SK snRNP inhibitory partner HEXIM1, SEC components (ELL2, AFF1 and ENL), and transcription factors (NF-κB, NFAT-c1 and AP1). Resting polarized Th17 cells prepared from healthy donor naïve CD4+ T cells were stimulated or not with anti-CD3/anti-CD28 Dynabeads for the times shown prior to preparation of low salt and chromatin nuclear fractions. Thereafter, both fractions were subjected to Western blotting using primary antibodies towards the proteins shown. Immunoblotting for GAPDH, β-Actin and Lamin B1 were performed to serve as fraction and loading controls. (B), (C) and (D) Examination of the mRNA expression of P-TEFb, SEC and 7SK snRNP subunits using a publicly available bulk RNA-seq dataset (SRA accession SRP026389) prepared using human memory CD4+ T cells that had been activated or not through TCR co-stimulation with anti-CD3/anti-CD28 coated beads for the times shown. (TIF) [file ppat.1009581.s006.tif]

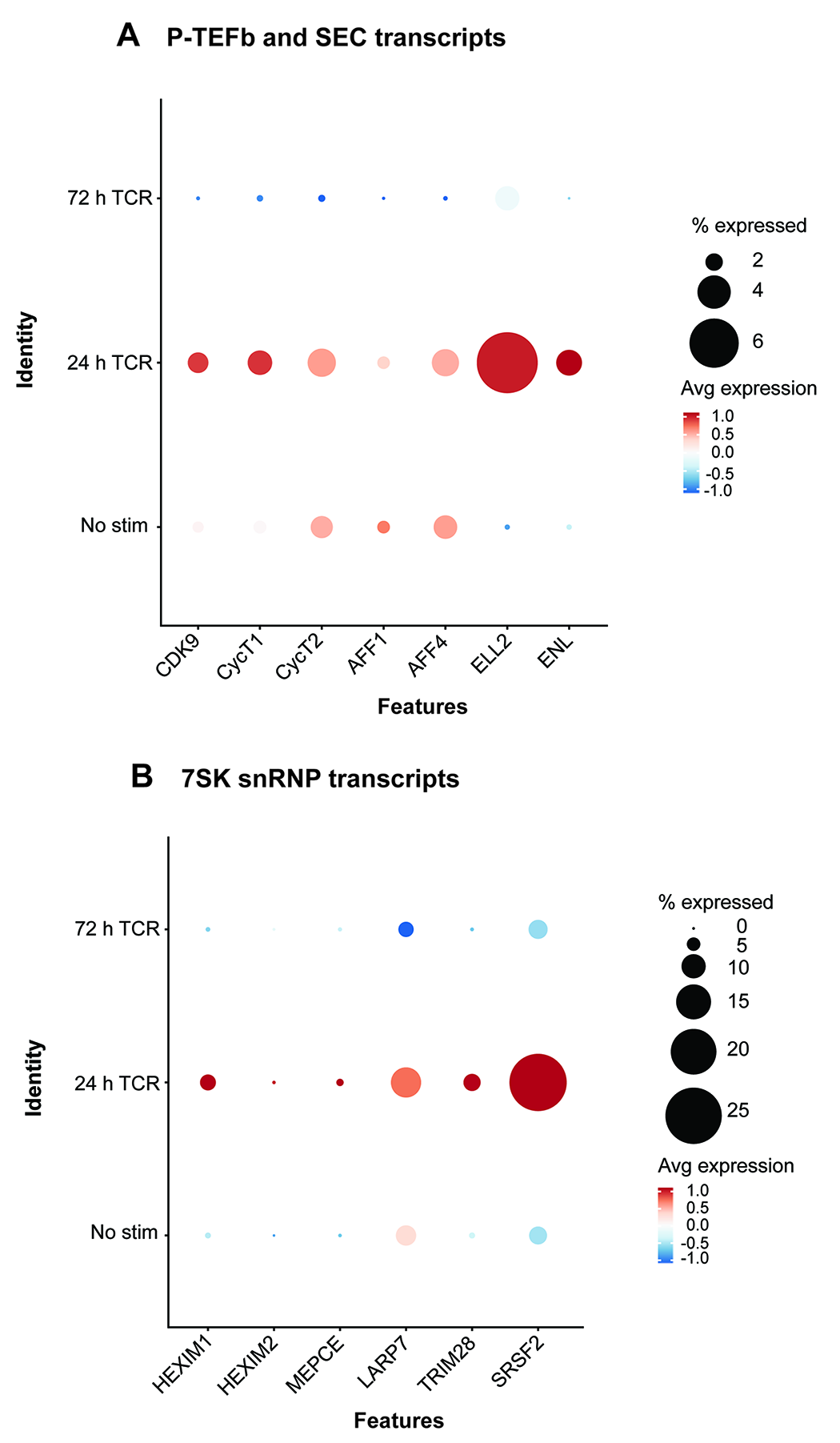

Supplement: S7 Fig — (A) and (B) Healthy donor-derived memory CD4+ T cells were allowed to recover overnight in complete RPMI medium with no IL-2 addition prior to challenging them or not with anti-CD3/anti-CD28 Dynabeads for 24 or 72 h at a 1:1 bead-to-cell ratio in media containing 15 IU/ml IL-2. Thereafter, cells were prepared for Drop-seq by magnetically removing the T-cell receptor activator beads, removal of dead cells using the MACS dead cell removal kit and resuspension of cells in 1X PBS containing 0.1% BSA at 2 X 105 cells/ml. Cells were clustered according to their unbiased transcriptome signatures (unsupervised clustering) and the dot plot function in Seurat was used to quantitate positive cell enrichment and relative expression levels of P-TEFb, SEC and 7SK snRNP transcripts in the dataset. (TIF) [file ppat.1009581.s007.tif]

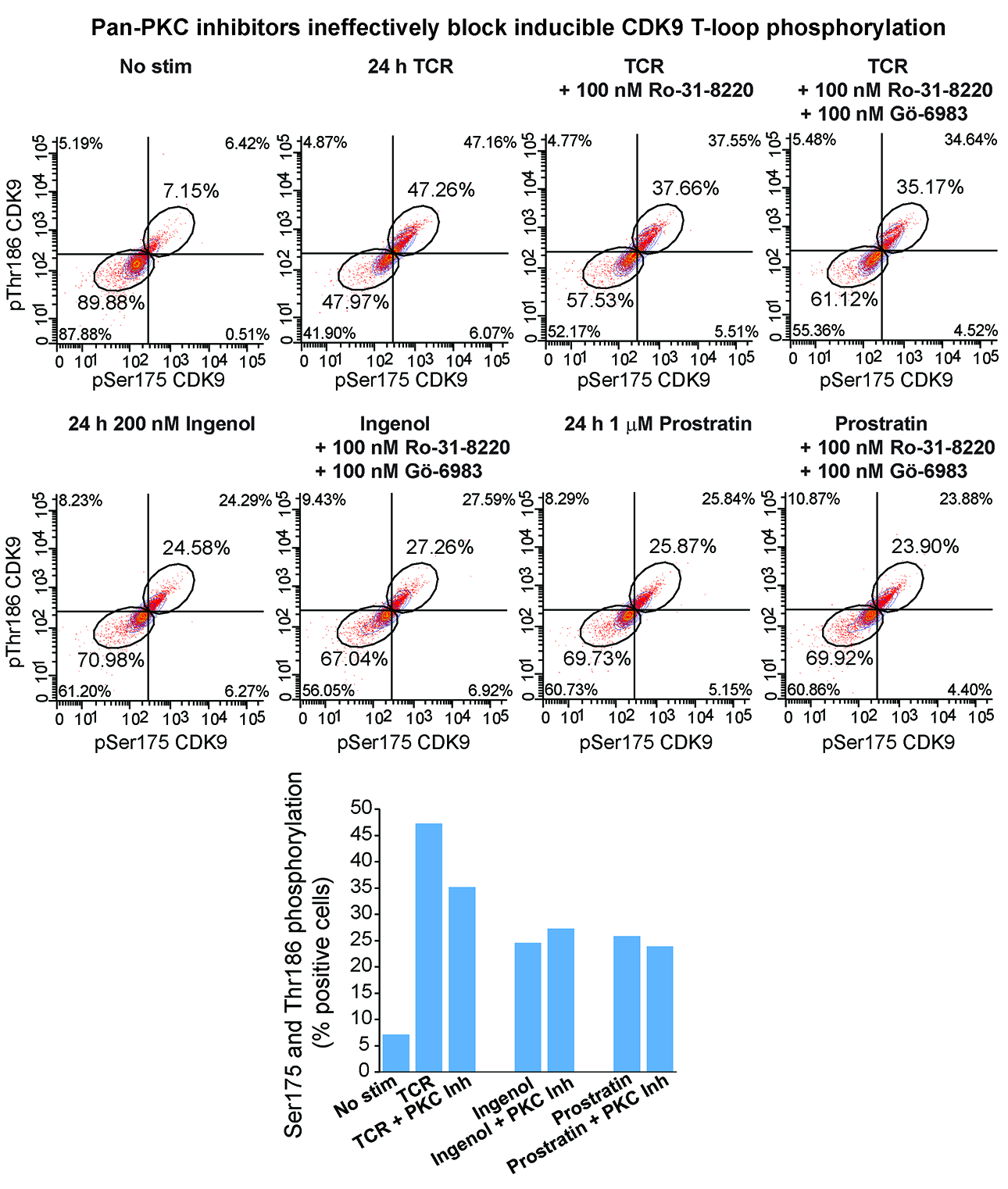

Supplement: S8 Fig — Cells were treated or not for 30 min with a combination of Ro-31-8220 and Gö 6983 at 100 nM each prior to stimulation with anti-CD3/anti-CD28 Dynabeads or challenge with ingenol or prostratin at the concentrations shown. Afterwards, cells were analyzed by flow cytometry following immunostaining using fluorophore-conjugated antibodies towards pThr186 CDK9 and pSer175 CDK9. The graph at the bottom is a representation of the flow cytometry data and shows the percentage of cells that are positive for both pSer175 and pThr186 CDK9. (TIF) [file ppat.1009581.s008.tif]

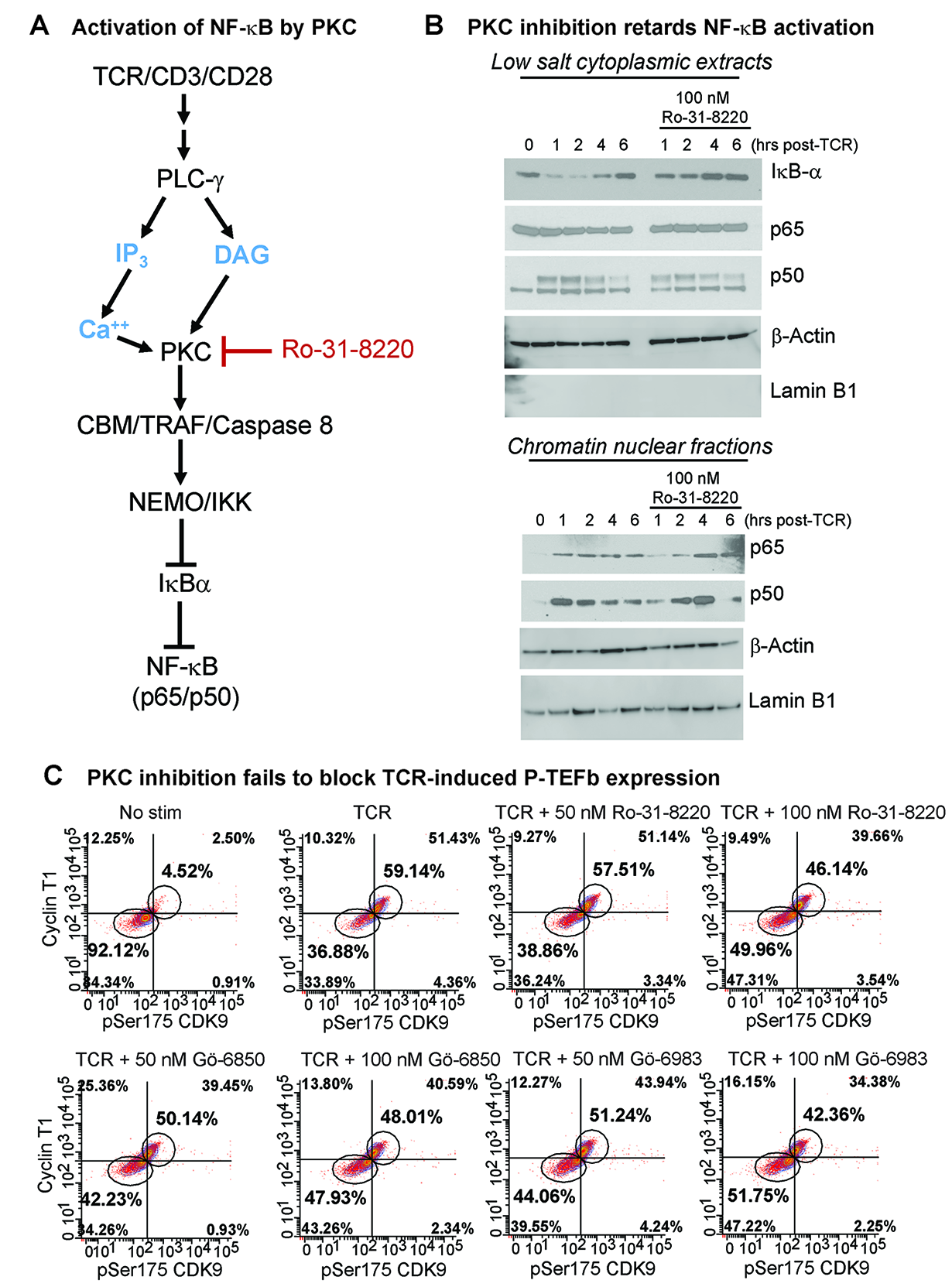

Supplement: S9 Fig — (A) Scheme for the activation of the transcription factor NF-κB by PKC in primary T cells upon TCR co-stimulation. Shown in red is the PKC inhibitor used in the subsequent experiments. (B) Immunoblotting analysis of memory CD4+ T-cell extracts demonstrating that the pan-PKC inhibitor Ro-31-8220 can retard the degradation on IκB-α and the nuclear induction of NF-κB. Healthy donor memory CD4+ T cells were treated or not with 100 nM Ro-31-8220 for 30 min prior to stimulation with anti-CD3/anti-CD28 Dynabeads for the times shown. Thereafter, low salt and chromatin nuclear fractions were prepared and subjected to Western blotting using primary antibodies towards IκB-α and NF-κB subunits p50 and p65. Extracts were also immunoblotted for β-actin and the nuclear membrane protein Lamin B1, which served as fractionation and loading controls. (C) PKC inhibitors are unable to suppress the induction of active P-TEFb in memory CD4+ T cells. Cells were treated or not for 30 min with Ro-31-8220, Gö 6950, or Gö 6983 at 100 nM each prior to TCR co-stimulation. Thereafter, cells were analyzed by flow cytometry following immunostaining using fluorophore-conjugated antibodies towards CycT1 and pSer175 CDK9. (TIF) [file ppat.1009581.s009.tif]

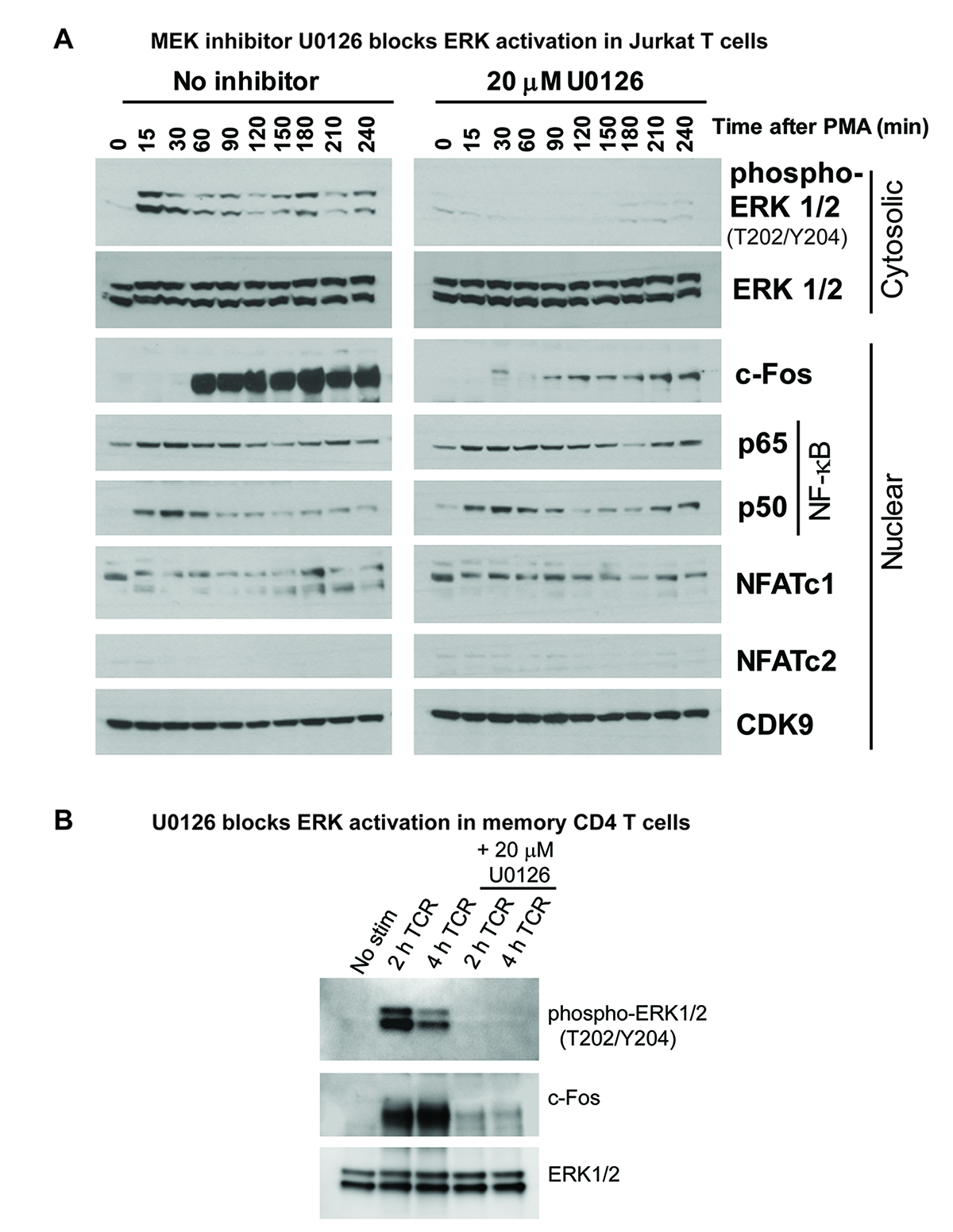

Supplement: S10 Fig — (A) Kinetic analysis of the activation of the MAP kinase isoforms ERK1 and ERK2, and the nuclear mobilization of c-Fos and NF-κB in response to PMA treatment with or without pretreatment with the MEK inhibitor U0126. Cytosolic and nuclear extracts were prepared from Jurkat T cells that were challenged or not with PMA for the indicated times in the presence or absence of U0126. These extracts were subjected to Western blotting to examine the expression of ERK1/2 and their phosphorylation by MEK, inducible expression of the transcription factor c-Fos, and the nuclear induction of c-Fos, NF-κB and NFAT proteins. Immunoblotting of cytosolic and nuclear extracts for ERK1/2 and CDK9, respectively, served as the loading controls in this experiment. (B) TCR activation of ERK1/2 phosphorylation by MEK in memory CD4+ T cells and subsequent ERK regulation of c-Fos are effectively blocked by U0126. Whole cell extracts were prepared from healthy donor memory CD4+ T cells stimulated or not through the TCR with anti-CD3/anti-CD28 Dynabeads for the times shown. These extracts were subjected to Western blotting to examine the expression of ERK1/2 and their phosphorylation by MEK, and inducible expression of the transcription factor c-Fos. Immunoblotting of the whole cell extracts for ERK1/2 served as the loading control in this experiment. (TIF) [file ppat.1009581.s010.tif]

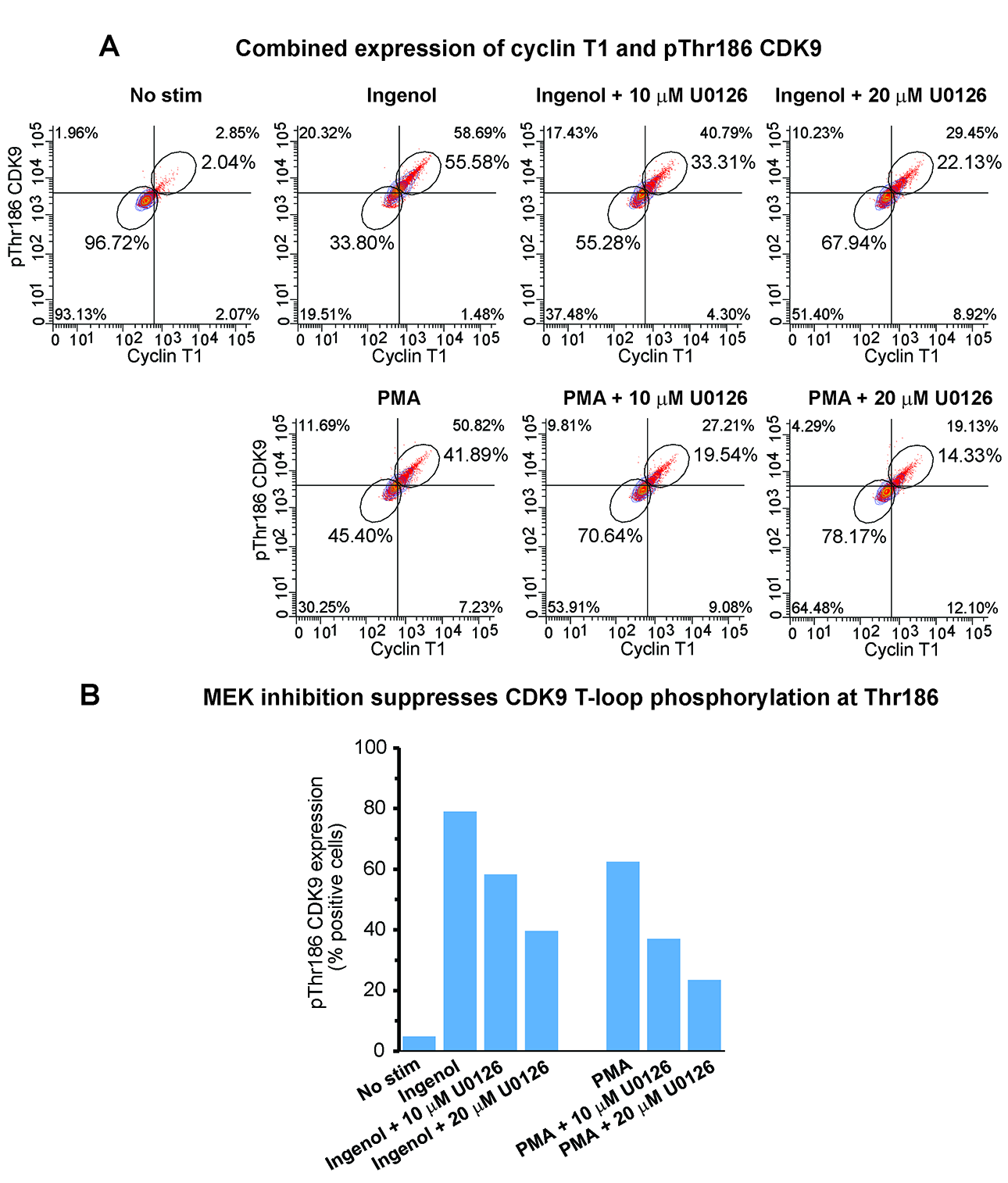

Supplement: S11 Fig — (A) Healthy donor memory CD4+ T cells were treated or not for 30 min with U0126 prior to 24 h TCR co-stimulation or challenge with ingenol or PMA. Afterwards, cells were analyzed by flow cytometry for cyclin T1 and pThr186 CDK9 expression following immunostaining using fluorophore-conjugated antibodies towards cyclin T1 and pThr186 CDK9. (B) The graph shows the percentage of cells that are positive for the expression of pThr186 CDK9. (TIF) [file ppat.1009581.s011.tif]

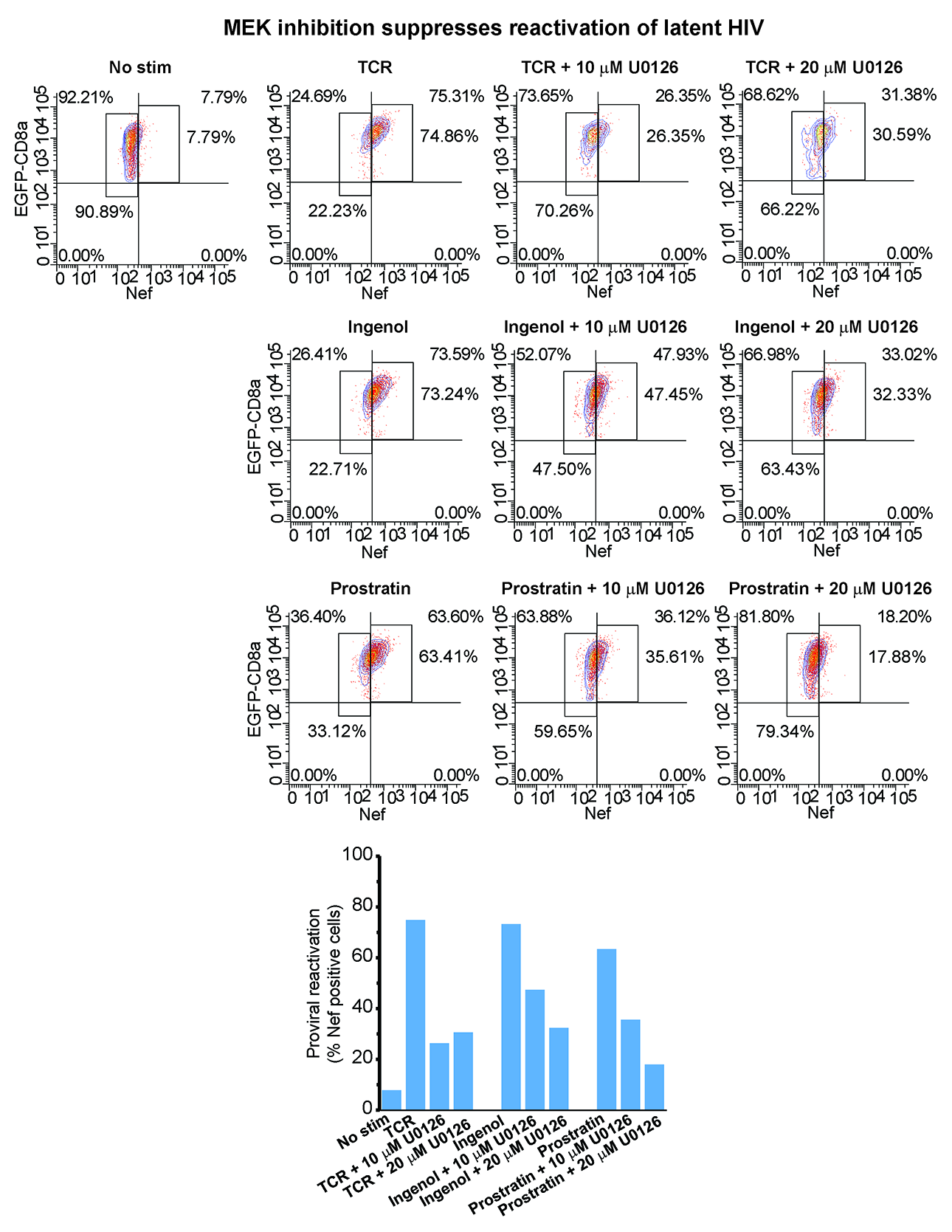

Supplement: S12 Fig — Latently infected resting primary Th17 cells were treated or not for 30 min with U0126 prior to 24 h TCR co-stimulation or challenge with the PKC agonists ingenol, prostratin or PMA. Thereafter, cells were analyzed by flow cytometry following immunostaining using a fluorophore-conjugated antibody towards HIV Nef. The graph at the bottom is a representation of the flow cytometry data and shows the percentage of cells that are positive for Nef expression. (TIF) [file ppat.1009581.s012.tif]

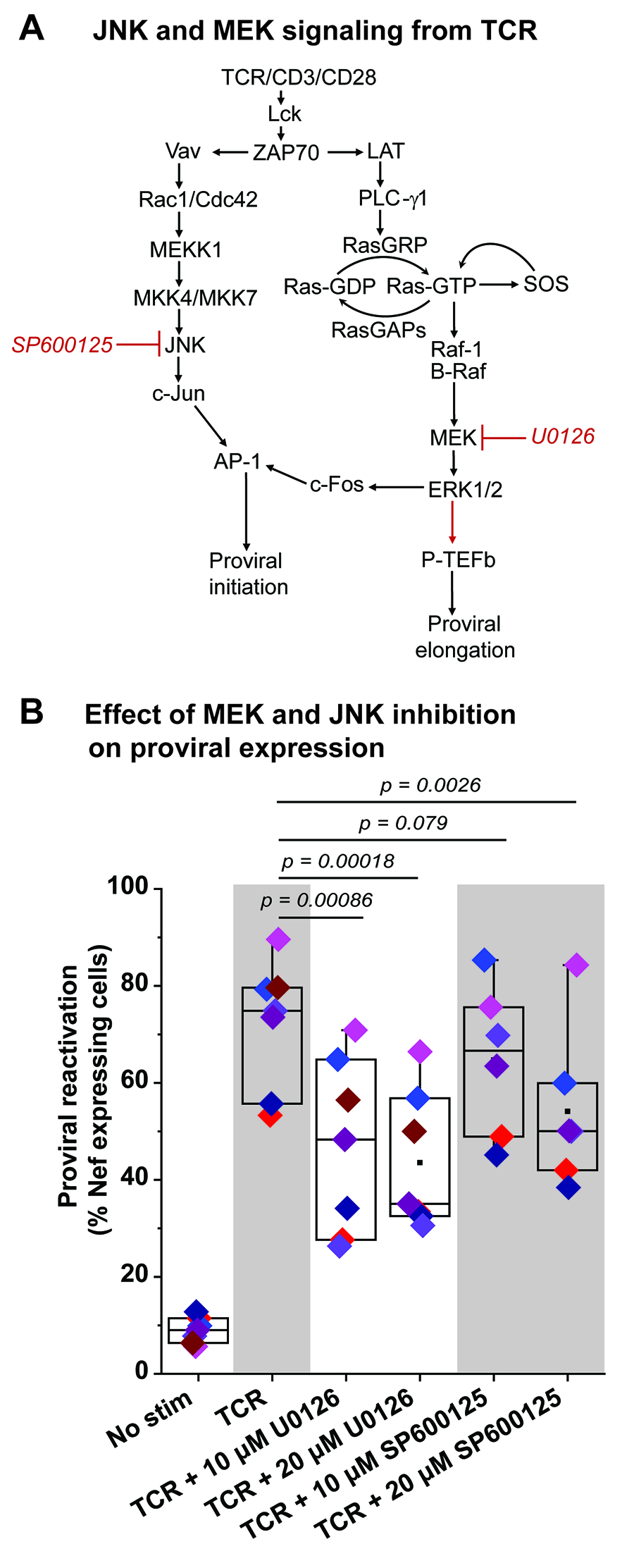

Supplement: S13 Fig — (A) Proposed scheme for the regulation of proviral reactivation by ERK and JNK MAPK signaling in TCR-activated primary T cells. (B) U0126 performs better than the JNK inhibitor SP600125 at suppressing the reactivation of latent HIV by TCR co-stimulation in HIV-infected Th17 cells albeit with noticeably significant variability. Latently infected primary Th17 cells prepared using naïve CD4+ T cells from three different donors were treated or not with U0126 or SP600125 for 30 min prior to TCR co-stimulation with anti-CD3/anti-CD28 Dynabeads for 24 h. Cells were then analyzed by flow cytometry following immunostaining using a fluorophore-conjugated antibody towards HIV Nef. The p values shown were calculated using a two-tailed Student’s t test. (TIF) [file ppat.1009581.s013.tif]

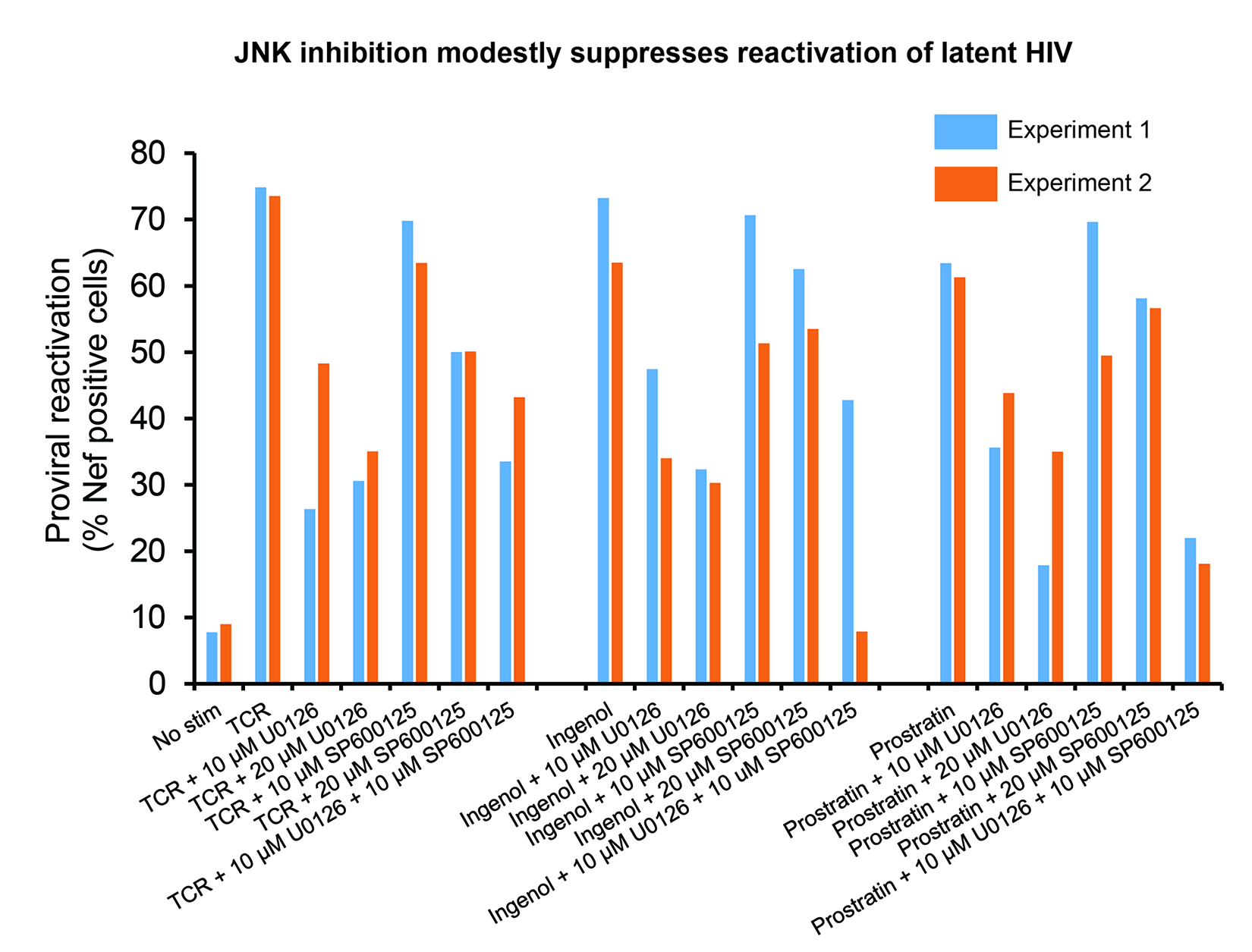

Supplement: S14 Fig — (A) Latently infected Th17 cells prepared using healthy donor naïve CD4+ T cells were treated or not for 30 min with either the MEK inhibitor U0126 or the JNK inhibitor SP600125 on their own or in combination prior to 24 h TCR co-stimulation or challenge with either ingenol or prostratin. Afterwards, cells were analyzed by flow cytometry following immunostaining using a fluorophore-conjugated antibody towards HIV Nef. The graph shows data from two separate experiments. (TIF) [file ppat.1009581.s014.tif]

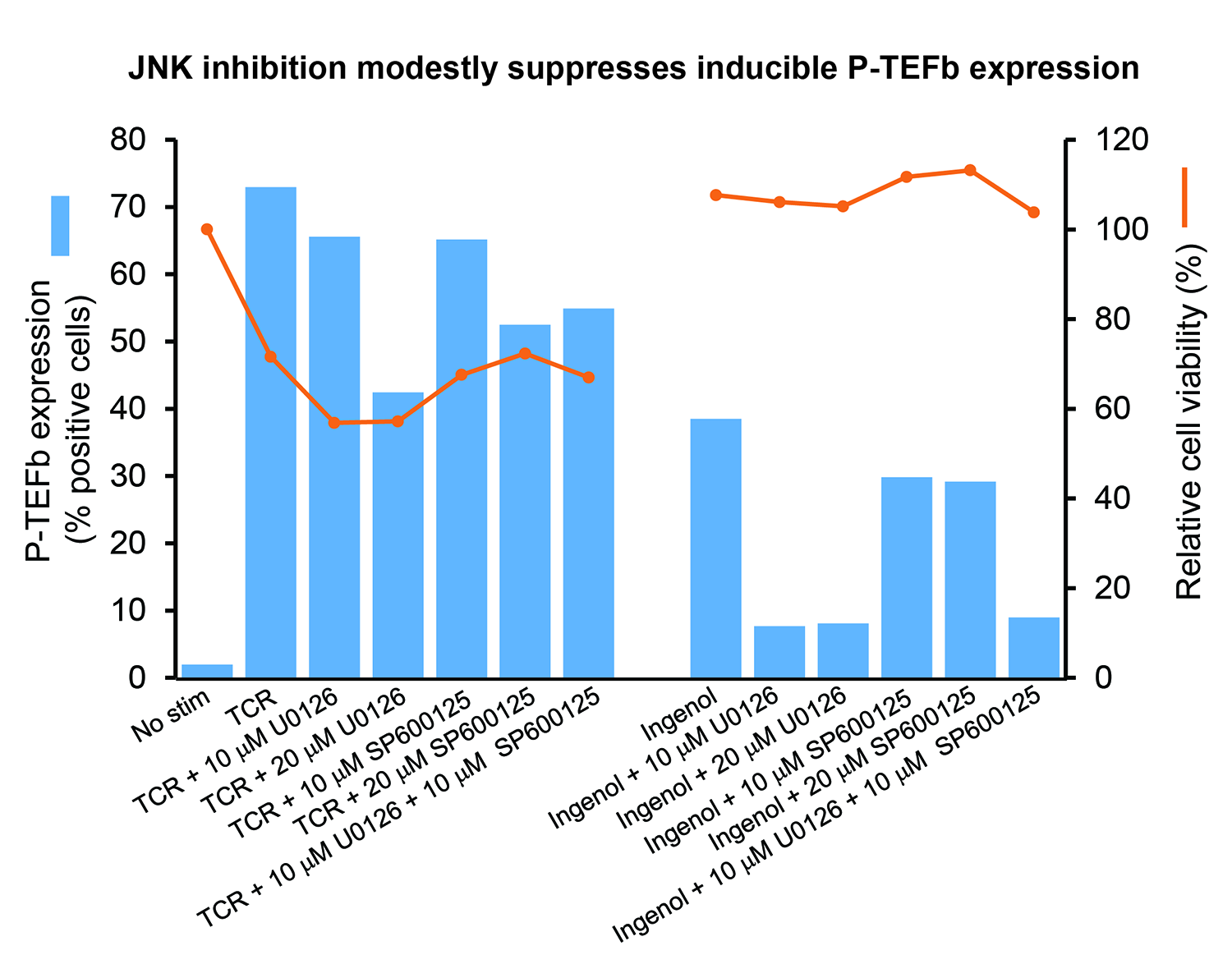

Supplement: S15 Fig — Healthy donor memory CD4+ T cells were treated or not for 30 min with either the MEK inhibitor U0126 or the JNK inhibitor SP600125 on their own or in combination prior to 24 h TCR co-stimulation or challenge with 50 nM ingenol. Afterwards, cells were analyzed by flow cytometry for active P-TEFb expression following immunostaining using fluorophore-conjugated antibodies towards cyclin T1 and pSer175 CDK9. Cell viability was also assessed by propidium iodide staining. The viability data shown are expressed as a percentage relative to non-treated cells. (TIF) [file ppat.1009581.s015.tif]

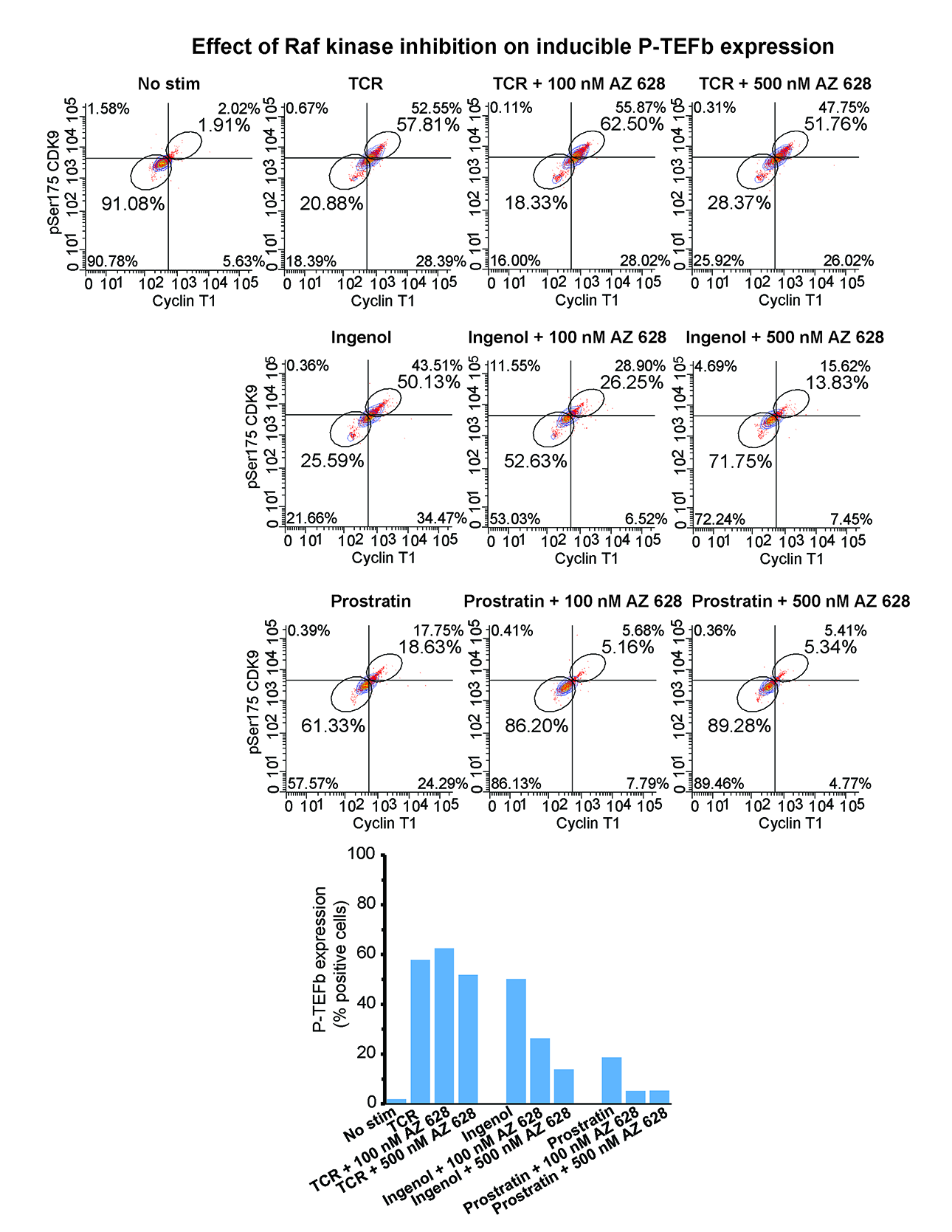

Supplement: S16 Fig — Healthy donor memory CD4+ T cells were treated or not for 30 min with AZ 628 at the concentrations shown prior to 24 h TCR co-stimulation or challenge with 50 nM ingenol or 1 μM prostratin. Afterwards, cells were analyzed by flow cytometry for active P-TEFb expression following immunostaining using fluorophore-conjugated antibodies towards CycT1 and pSer175 CDK9. The graph at the bottom is a representation of the flow cytometry data and shows the percentage of cells that are positive for both CycT1 and pSer175 CDK9. (TIF) [file ppat.1009581.s016.tif]

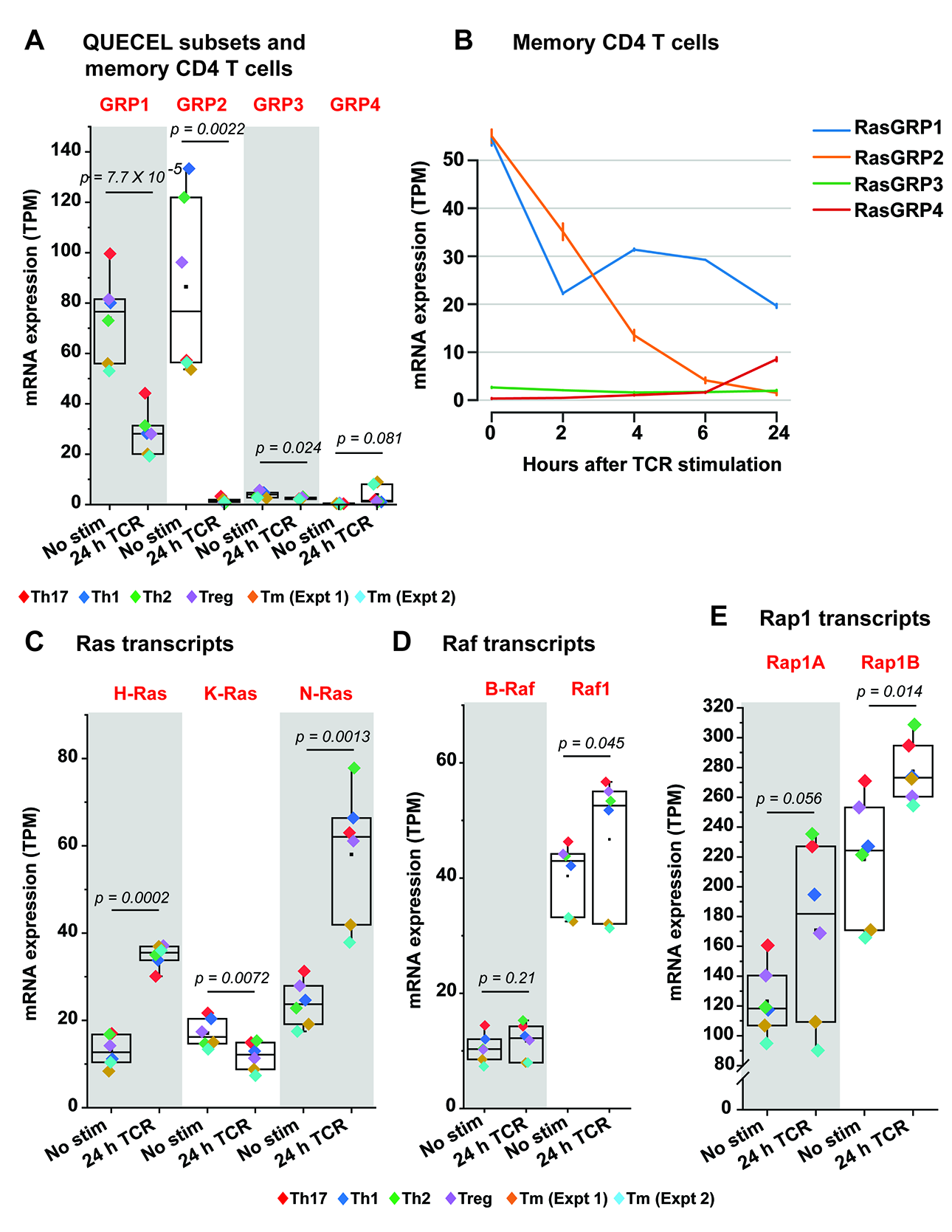

Supplement: S17 Fig — Quiescent CD4+ T cells that had been polarized into Th1, Th2, Treg and Th17 subsets using the QUECEL procedure were activated or not for 24 h with anti-CD3/anti-CD28 Dynabeads. Bulk RNA-seq datasets obtained using these cells were analyzed to examine the expression of RasGRP (A), Ras (C), Raf (D) and Rap1 (E) isoforms. A publicly available bulk RNA-seq dataset of primary human memory CD4+ T cells that had been activated or not through TCR co-stimulation with anti-CD3/anti-CD28 coated beads (SRA accession SRP026389) was also analyzed for these factors (Tm Expt 1 and Tm Expt 2). In B, additional assessment of RasGRP isoform expression was performed over the entire time course of TCR activation (0, 2, 4, 6, and 24 h) for which the memory T-cell bulk RNA-seq dataset was generated. Transcripts per million (TPM) values were used to evaluate the relative abundance of transcripts under resting and TCR-activated conditions. Statistical significance (p values) was calculated using a two-tailed Student’s t test. (TIF) [file ppat.1009581.s017.tif]

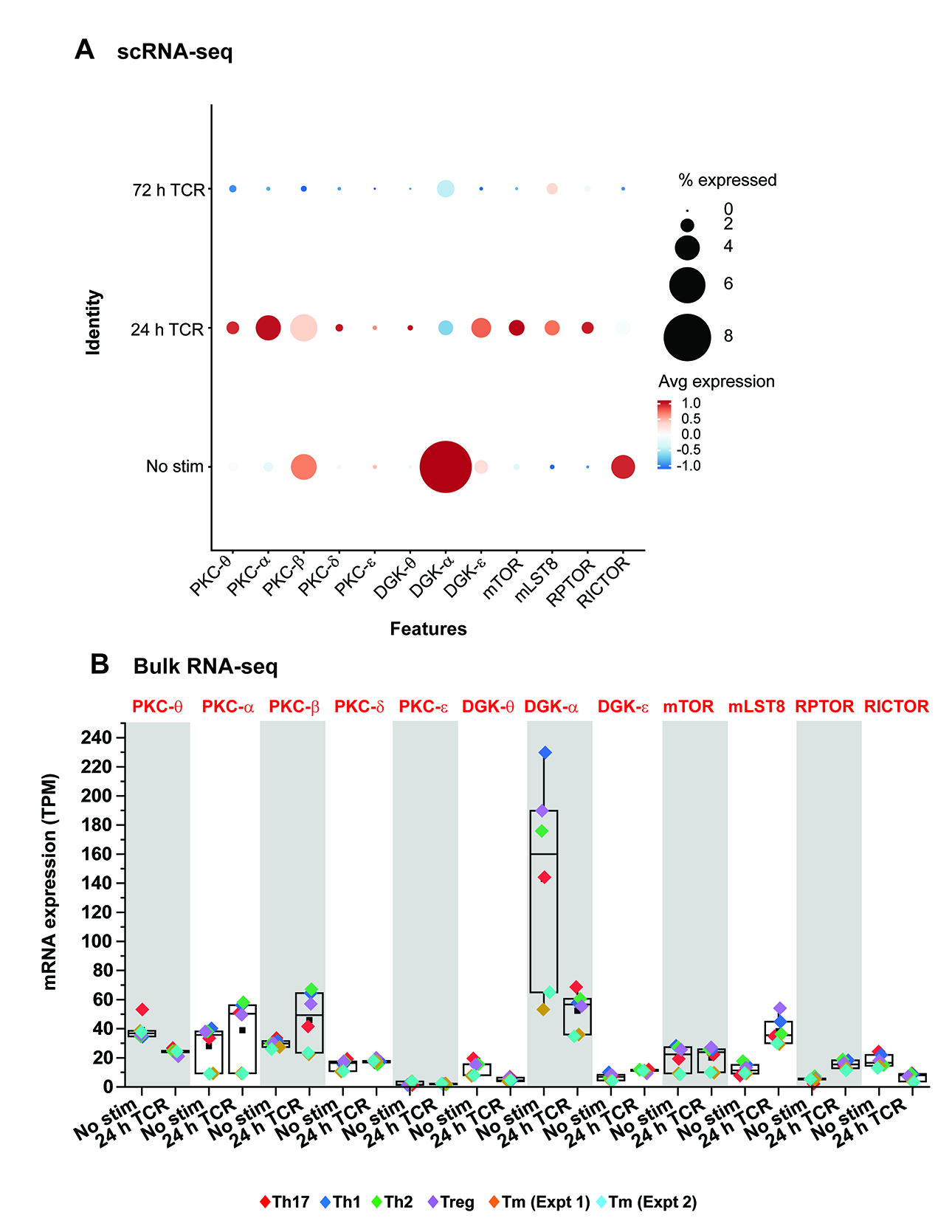

Supplement: S18 Fig — (A) scRNA-seq analysis of unstimulated and TCR-activated memory CD4+ T cells. Healthy donor-derived memory CD4+ T cells were stimulated or not with anti-CD3/anti-CD28 Dynabeads for 24 or 72 h prior to being subjected to Drop-seq. Cells were clustered according to their unbiased transcriptome signatures (unsupervised clustering) and the dot plot function in Seurat was used to quantitate positive cell enrichment and relative expression levels of PKC, DGK and mTORC transcripts in the dataset. (B) Bulk RNA-seq analysis of primary QUECEL subsets and memory CD4+ T cells. Quiescent CD4+ T cells that had been polarized into Th1, Th2, Treg and Th17 subsets using the QUECEL procedure were activated or not for 24 h with anti-CD3/anti-CD28 Dynabeads. Bulk RNA-seq datasets obtained using these cells were analyzed to examine the expression of PKC, DGK and mTORC transcripts. A publicly available bulk RNA-seq dataset of primary human memory CD4+ T cells that had been activated or not through TCR co-stimulation with anti-CD3/anti-CD28 coated beads (SRA accession SRP026389) was also included in the analysis (Tm Expt 1 and Tm Expt 2). (TIF) [file ppat.1009581.s018.tif]

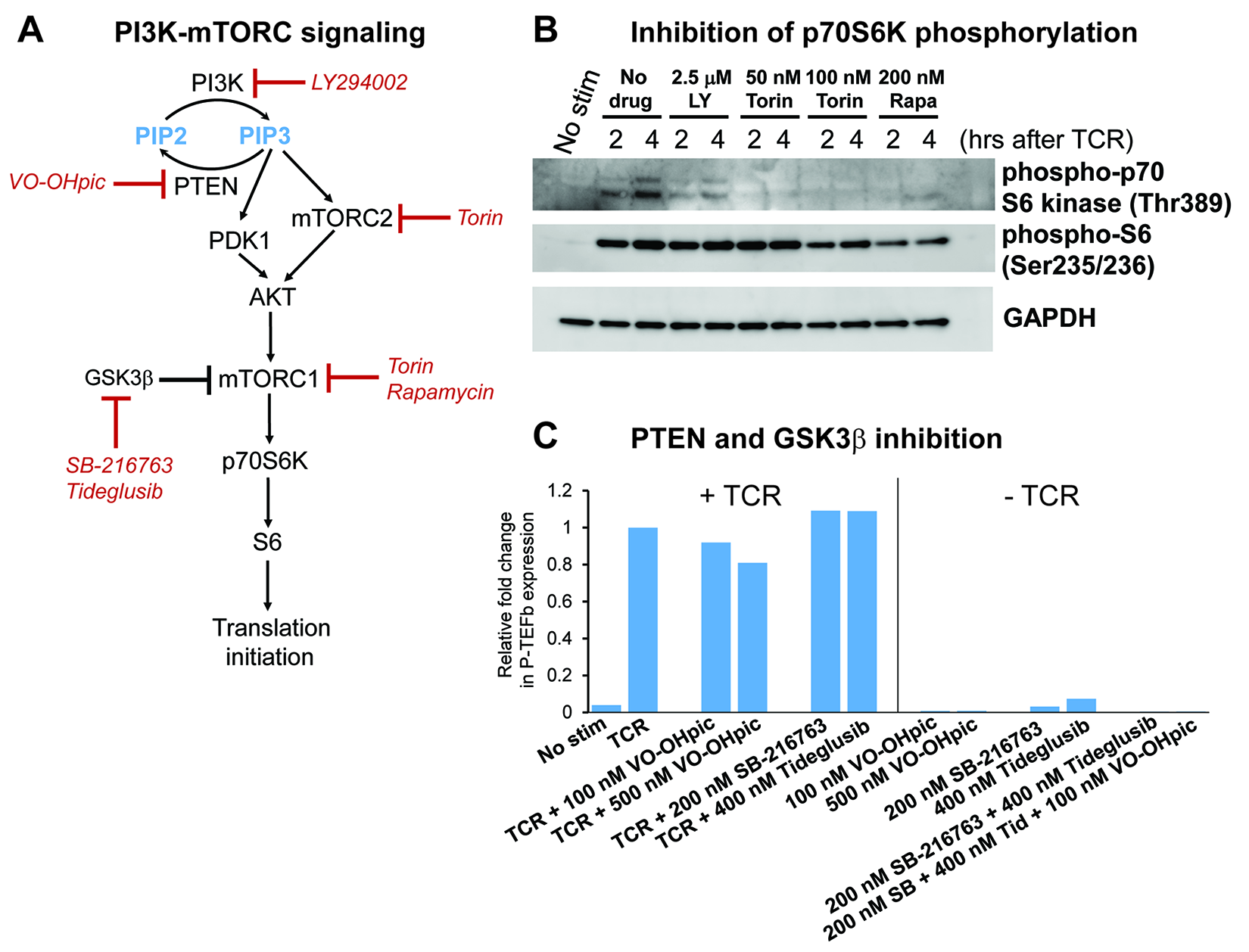

Supplement: S19 Fig — (A) Scheme for PI3K-AKT-mTORC signaling showing the inhibitors tested in B and C. (B) PI3K and mTORC inhibitors effectively block p70 S6 kinase (p70S6K) phosphorylation at the concentrations employed in the current study. Whole cell extracts were prepared from memory T cells treated or not with LY294002, Torin or rapamycin at the concentrations shown prior to TCR activation for 2 or 4 h. Thereafter, Western blotting was performed to examine the expression of phospho-p70S6K and phospho-S6. Immunoblotting for GAPDH served as a loading control in this experiment. (C) Effect of inhibiting GSK3β and PTEN on P-TEFb expression in memory T cells. Cells were treated or not for 30 min with inhibitors towards PTEN (VO-OHpic) or GSK3β (SB216763 or Tideglusib) at the concentrations shown prior 24 h TCR co-stimulation. Alternatively, cells were treated with these inhibitors on their own or in combination as shown. Afterwards, cells were analyzed by flow cytometry for active P-TEFb expression following immunostaining using fluorophore-conjugated antibodies towards CycT1 and pSer175 CDK9. The vertical axis shows the fold change in P-TEFb expression relative to the TCR activation condition. (TIF) [file ppat.1009581.s019.tif]

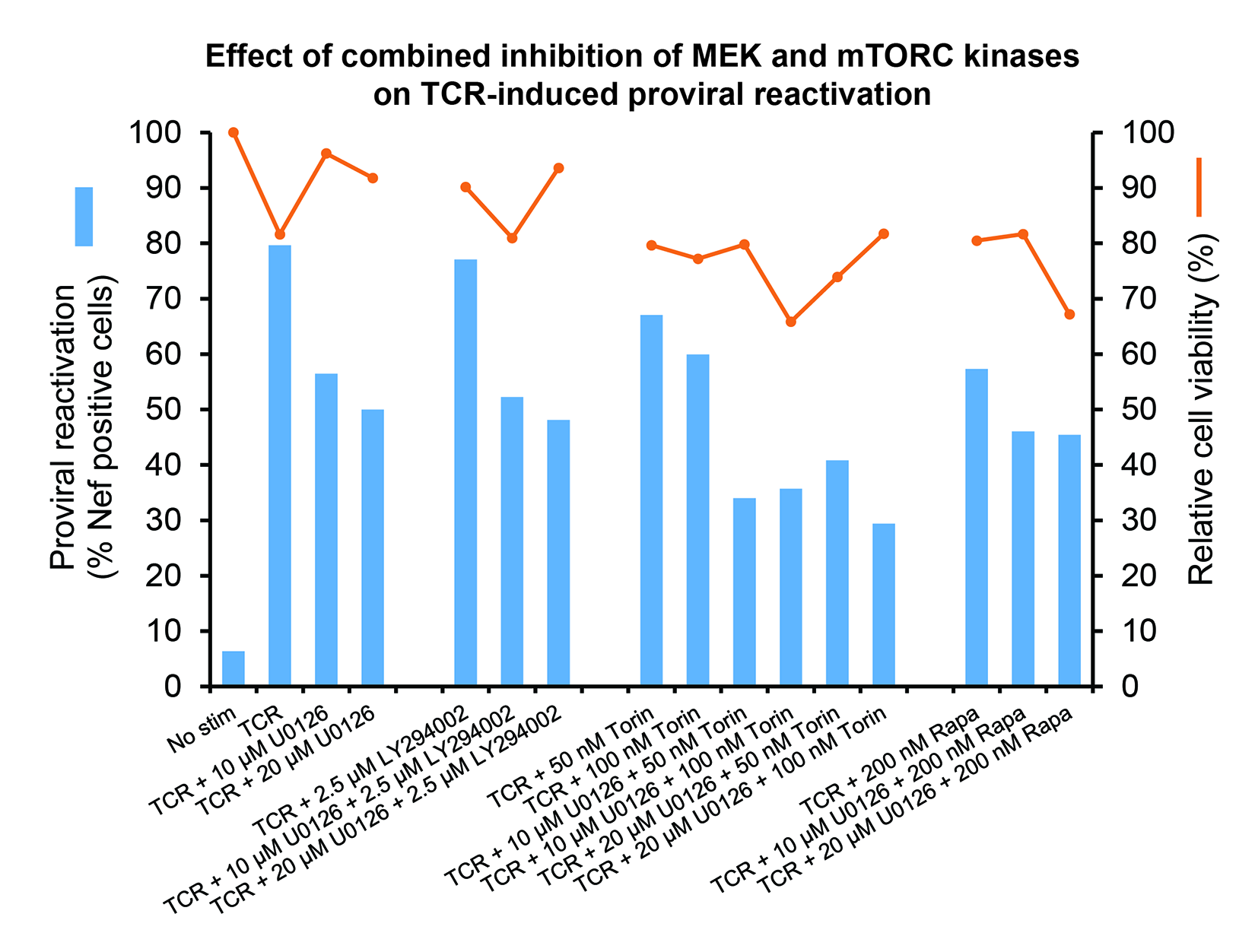

Supplement: S20 Fig — Latently infected Th17 cells prepared using naïve CD4+ T cells isolated from a healthy donor were pretreated or not for 30 min with the inhibitors shown prior to TCR co-stimulation for 24 h. Afterwards, cells were analyzed by flow cytometry following immunostaining using a fluorophore-conjugated antibody towards HIV Nef. Cell viability was also assessed by flow cytometry following staining with the eFluor450 viability dye. The viability data shown are expressed as a percentage relative to non-treated cells. (TIF) [file ppat.1009581.s020.tif]

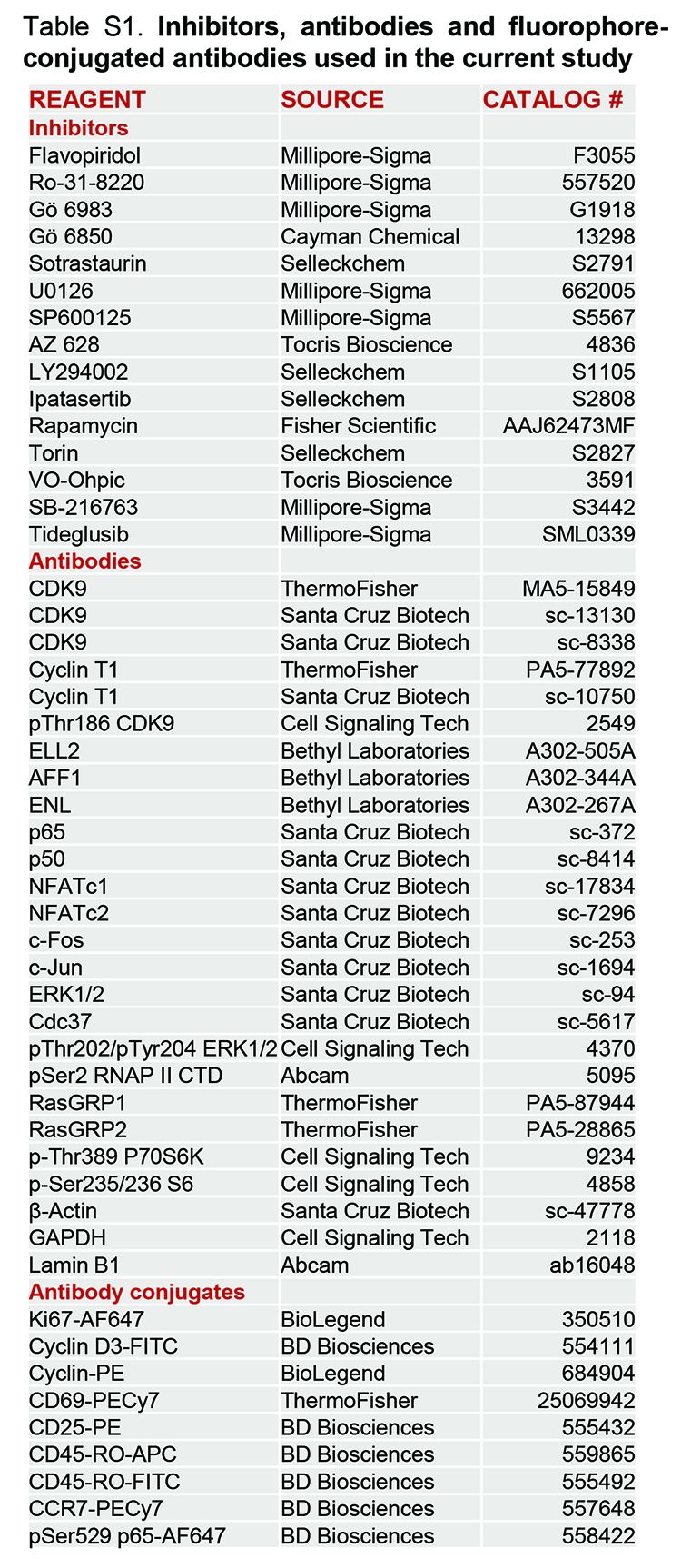

Supplement: S1 Table — (TIF) [file ppat.1009581.s021.tif]

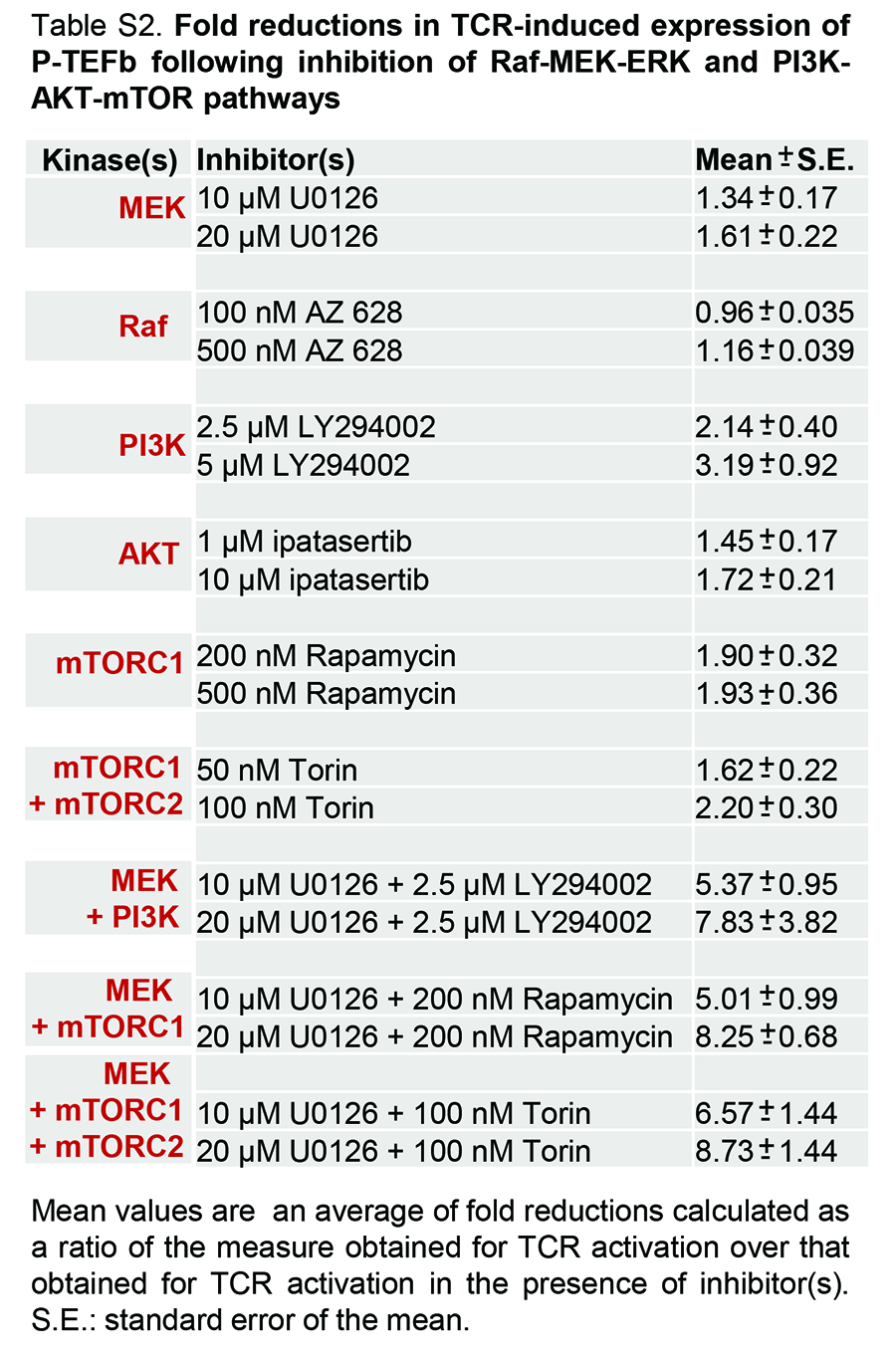

Supplement: S2 Table — (TIF) [file ppat.1009581.s022.tif]
